# Tracing down the Updates on Dengue Virus—Molecular Biology, Antivirals, and Vaccine Strategies

**DOI:** 10.3390/vaccines11081328

**Published:** 2023-08-05

**Authors:** Shiza Malik, Omar Ahsan, Hassan Mumtaz, Muhammad Tahir Khan, Ranjit Sah, Yasir Waheed

**Affiliations:** 1 Bridging Health Foundation, Rawalpindi 46000, Pakistan; 2 Department of Medicine, Foundation University Medical College, Foundation University Islamabad, Islamabad 44000, Pakistan; 3 Innovation, Implementation, and Partnership Unit, Association for Social Development, Islamabad 44000, Pakistan; 4 Health Services Academy, Islamabad 44000, Pakistan; 5 Institute of Molecular Biology and Biotechnology (IMBB), University of Lahore, 1KM Defence Road, Lahore 58810, Pakistan; 6 Zhongjing Research and Industrialization Institute of Chinese Medicine, Zhongguancun Scientific Park, Meixi, Nanyang 473006, China; 7 Department of Microbiology, Tribhuvan University Teaching Hospital, Institute of Medicine, Kathmandu 44600, Nepal; 8 Department of Microbiology, Dr. D. Y. Patil Medical College, Hospital and Research Centre, Dr. D. Y. Patil Vidyapeeth, Pune 411018, Maharashtra, India; 9 Department of Public Health Dentistry, Dr. D.Y. Patil Dental College and Hospital, Dr. D.Y. Patil Vidyapeeth, Pune 411018, Maharashtra, India; 10 Office of Research, Innovation and Commercialization (ORIC), Shaheed Zulfiqar Ali Bhutto Medical University (SZABMU), Islamabad 44000, Pakistan; 11 Gilbert and Rose-Marie Chagoury School of Medicine, Lebanese American University, Byblos 1401, Lebanon

**Keywords:** dengue virus (DENV), dengue fever (DF), vaccines, antiviral targets, drugs, therapeutic approaches, ADE

## Abstract

Background: Nearly half of the world is at risk of developing dengue infection. Dengue virus is the causative agent behind this public healthcare concern. Millions of dengue cases are reported every year, leading to thousands of deaths. The scientific community is working to develop effective therapeutic strategies in the form of vaccines and antiviral drugs against dengue. Methods: In this review, a methodological approach has been used to gather data from the past five years to include the latest developments against the dengue virus. Results: Different therapeutics and antiviral targets against the dengue virus are at different stages of development, but none have been approved by the FDA. Moreover, various vaccination strategies have also been discussed, including attenuated virus vaccines, recombinant subunit vaccines, viral vector vaccines, DNA vaccines, nanotechnology, and plant-based vaccines, which are used to develop effective vaccines for the dengue virus. Many dengue vaccines pass the initial phases of evaluation, but only two vaccines have been approved for public use. DENGVAXIA is the only FDA-approved vaccine against all four stereotypes of the dengue virus, but it is licensed for use only in individuals 6–16 years of age with laboratory-confirmed previous dengue infection and living in endemic countries. Takeda is the second vaccine approved for use in the European Union, the United Kingdom, Brazil, Argentina, Indonesia, and Thailand. It produced sustained antibody responses against all four serotypes of dengue virus, regardless of previous exposure and dosing schedule. Other dengue vaccine candidates at different stages of development are TV-003/005, TDENV PIV, V180, and some DNA vaccines. Conclusion: There is a need to put more effort into developing effective vaccines and therapeutics for dengue, as already approved vaccines and therapeutics have limitations. DENGVAXIA is approved for use in children and teenagers who are 6–16 years of age and have confirmed dengue infection, while Takeda is approved for use in certain countries, and it has withdrawn its application for FDA approval.

## 1. Introduction

Dengue virus (DENV) has been known for a long time to cause dengue fever (DF), dengue hemorrhagic fever (DHF), and dengue shock syndrome (DSS) with lethal outcomes, causing a public health concern for many years [1]. The healthcare burden that emerges from the dengue endemic has heavily impacted the medical, healthcare, and economic standing of affected endemic regions. Developing countries are especially affected because of poor environmental conditions that increase the chances of the virus spreading [2]. Although it may be treated as a neglected tropical disease, it leads to millions of dengue cases and thousands of deaths every year. Statistical data from WHO indicate that the global incidence of dengue virus has grown dramatically and has put half of the world’s population at risk of developing DF [3]. An estimated 100–400 million people annually become dengue infection; though 80% remain mild and asymptomatic, 20% develop severe DF, leading to ~22,000 deaths annually [4,5].

DENV belongs to the Flaviviridae family of viruses. Structurally, it is composed of a positive-stranded RNA genome. It has been classified into four to five different serotypes worldwide that may slightly differ in antigenicity from each other. There are multiple genetic variants within each DENV serotype and even in each viral isolate of an RNA-containing viral quasispecies [3,6]. If a person gets infected with any of these serotypes, they may develop immunity against that particular serotype along with short-term immunity against other serotypes. This aspect is really important for scientists working on antiviral drugs and vaccines against the dengue virus [2]. Clinical manifestations of dengue infection (caused by different serotypes) are diverse. These may include mild fever to severe dengue infections in the forms of DHF and DSS [2,7]. Other symptoms may include a severe headache, abdominal pain, skin rash, muscular fatigue, joint pain, loss of appetite, vomiting, diarrhea, and an unpleasant taste. Additionally, the viral infection may produce impaired physical and cognitive development [2,8].

The persistent endemic nature of dengue is now slowly transforming into an epidemic status owing to the globalization of the healthcare burden of infectious diseases. Approximately more than 100 countries in regions of Africa, Asia, America, the Middle East, and the Western Pacific have been identified where dengue is an aggressively spreading disease [6,9]. The mild and asymptomatic nature of dengue infection makes it less reported throughout these regions. However, it is counted among the most rapidly spreading diseases caused by mosquitoes after malaria. Specific genera of *Aedes* mosquito (*Aedes aegypti* and *Aedes albopictus*) cause viral transmission and life cycle progression [6].

Third-world countries have to bear the burden of neglected tropical diseases in the form of deprivation of schooling for children, job insecurity, financial burden, and healthcare compromise, among others [10]. The severity of the healthcare burden linked to dengue infection makes it imperative for scientists to bring about a medical solution to the disease. Broad-scale research studies have been conducted in this regard, and some antiviral agents and vaccination protocols have been undergoing experimental phases for approval against dengue infection [6,10]. In this article, we review some of the latest developments in vaccinology and the pharmaceutical domain regarding the therapeutic trail of dengue virus disease. The discussion will help the scientific community to understand the best possible vaccine candidates that are and can be employed for dengue infection, and the data will help to delve into the understanding of various antiviral strategies that can be adopted for other related viral infectious diseases.

## 2. Methodology

In this review, we adopted a methodological approach for collecting data regarding vaccination and antiviral strategies for DENV. Data were gathered from relatable publications adopted from the platforms of Google Scholar, PubMed, and Web of Science, among others. Major search terms were dengue virus, dengue fever, vaccine strategies, antiviral therapies, and modern drugs against the virus. Note that we limited the data referenced in the past seven years (2015–2023) to include only the latest developments in the related field of interest. 

## 3. Results

### 3.1. History and Epidemiology of Dengue

The first dengue outbreak can be traced back to 1779, mainly in the regions of Asia and Europe. Later, a North American outbreak was reported in 1780. 2010 recorded 1.6 million affected dengue cases, with approximately 49,000 severe DHF cases. A more severe outbreak was observed later in 2016 in the USA, where ~2.4 million cases of DF were reported [3,6,10] In 2019, nearly 3 million new cases of dengue infection were documented. In Africa, several outbreaks of limited and larger scale have been reported over the past years. Moreover, five large-scale epidemics were recorded in different countries in Africa from 1977 to 2009 [1,11].

Dengue virus is still resisting and showing reverberating occurrences in the form of endemics in different regions, mainly spreading out to vulnerable regions with compromised healthcare systems, yet also leading to infection in developed nations [1]. From the 1950s until recent years, DENV has been continuously showing emerging cases in different countries in Southeast Asia. The outbreaks in Southeast Asia date back to 1953, markedly as a consequential impact of urbanization and globalization following WWII. Indonesia suffered from the second-largest outbreak of dengue (2004–2010) after the Brazilian outbreak of dengue back in 2016 [12]. The recent outbreak of dengue (2021–present) in Asian countries has wreaked havoc on the healthcare system in developing nations, which are already suffering from the disease burden of the COVID-19 pandemic [6,13]. Corresponding to flavivirus circulation, multiple serotypes (so-called hyperendemnicity) are well known and are based on rearrangements of viral quasispecies [14]. Thus, it is imperative for the scientific community to understand and develop a vaccine approach against DENV infection.

### 3.2. Molecular Biology of DENV Infection

Two mosquito species named *Aedes aegypti* and *Aedes albopictus* are responsible for the transmission of the dengue virus. *Aedes aegypti* are largely endophilic, survive in water-filled spaces, and prefer daybiting. They are largely populated in tropical and subtropical areas of almost every continent [14,15]. *Aedes albopictus*, on the contrary, is exophilic, prefers living outdoors, and aggressively feeds on human blood. Any serotype transfer of DENV is the same and the whole process of transfer occurs mainly in two hosts, i.e., wild animals and humans. Although there are some other transmission cycles as well that run in parallel to other hosts like vertebrate species, here we will largely focus on DENV transmission from mosquitoes to animal reservoirs, where animal hosts are largely primates [6].

DENV undergoes successful infection through the bite of an *Aedes* mosquito and then primarily attacks different cells of the immune system, such as dendritic cells, macrophages, monocytes, and lymphocytes. These cells then undergo innate and adaptive immune responses [16]. Like various viral infections, entry of DENV is mediated through receptor binding and endocytosis through host membranous structures, such as FC receptors that bind to the constant fragment of immunoglobulins, lectin-like receptors, glycosaminoglycans (GAG), and lipopolysaccharide-binding molecules [13,17].

The viral nucleocapsid (NC) is subsequently released into the host cell cytoplasm upon acidification of the endosomes and rearrangement of E-proteins, which causes host and viral cell membrane fusion. This step releases the viral genome, which travels to the host endoplasmic reticulum (ER) by cytoplasmic transport machinery [11,18]. However, the replication machinery operates differently in the case of clathrin and receptor-mediated endocytosis, in which, after internalization of DENV, the virions remain trapped in endosomes and lysosomes and are not directly released in the cytoplasm. These processes need further research to be explained this in detail [19]. However, in general, at the ER, viral RNA is used as a template strand to translate into a polyprotein structure. The replicative complex of flaviviruses consists of viral (NS5, NS3, and other small nonstructural proteins) and cellular subunits. Both the structure and functions of flavivirus RNA-dependent RNA polymerase are well known [11,19,20]. During this translation, DENV genes and proteins (such as NS5) with cap methylation characteristics and RNA polymerase activity play a vital role in the replication process. The capsid proteins are translated in the host cytoplasm, while the E and M proteins of the virus are translated through ER membranous structures [18].

After repeated replication cycles and formation of sufficient positive RNA strands, the genome is encapsulated in capsid proteins within the cytoplasm of the host cell, and finally the virus buds off the host cell membrane (a reversal of viral entry and subsequent steps) [18]. During the budding off phase, it acquires E and M proteins on the surface with the help of host protease enzymes. Surface viral confirmation is pH-dependent. After proper cellular rearrangement and passage through the Golgi apparatus and the ER complex, the virus buds off with a second ER-driven outer membrane. The mature enveloped virus finally releases into the extracellular spaces to infect neighboring host cells and increase the viral load [11,18,19].

### 3.3. Quasispecies and Hyperendemnicity of Dengue Virus: A Challenge in Therapeutics and Vaccine Development

There are four distinct serotypes of dengue virus, known as DENV-1, DENV-2, DENV-3, and DENV-4. Each genotype can form its own quasispecial population due to the high mutation rate of the virus [1]. A quasispecies is a population of viruses or other microorganisms that have a high mutation rate and exist as a complex and diverse group of closely related variants [2,3]. Quasispecies is a term commonly used in virology to describe the genetic diversity observed within viral populations. Within each serotype, there can be multiple strains or variants of the virus that differ in their genetic makeup. These variants arise due to errors made during the replication process of the viral genome [11]. Quasispecies of dengue virus can also arise due to recombination events, where genetic material from different strains gets mixed and produces new variants. The presence of quasispecies in dengue virus populations has significant implications for disease outcome, transmission dynamics, and vaccine development [1,2,3]. The genetic diversity within the quasispecies can influence the severity of dengue disease because certain variants may be more virulent or have an increased capacity for immune evasion. Furthermore, the presence of diverse variants within the quasispecies can pose challenges for vaccine development [4,5,6]. Vaccines targeting one specific strain or serotype may not provide adequate protection against other variants within the quasispecies. Experts are continuously studying the dengue virus quasispecies to understand its impact on disease progression, transmission dynamics, and to develop effective strategies for prevention and control [7,8,9,10,11,12].

Similarly, hyperendemicity refers to the presence of multiple dengue stereotypes circulating in a specific area. In hyperendemic regions, individuals may be exposed to multiple serotypes throughout their lifetime, which can increase the risk of severe dengue [1,2,3,4]. In the case of dengue virus, hyperendemicity is characterized by the continuous circulation of multiple serotypes of the virus, resulting in frequent outbreaks and a heavy burden of disease. Dengue fever is caused by four closely related serotypes of the dengue virus (DEN-1, DEN-2, DEN-3, and DEN-4), which are transmitted to humans through the bite of infected *Aedes* mosquitoes [1,2,3,4,5,6]. Hyperendemic areas are typically tropical and subtropical regions where the *Aedes* mosquito thrives and the conditions for dengue transmission are favorable. The hyperendemicity of dengue poses several challenges for vaccine development. First, the presence of multiple serotypes means that an effective vaccine should provide protection against all of them. Vaccines must induce a strong and balanced immune response against each serotype because previous dengue infection with one serotype can lead to severe illness upon subsequent infection with a different serotype [6]. Secondly, the presence of multiple serotypes also increases the risk of antibody-dependent enhancement (ADE), a phenomenon where antibodies from a previous dengue infection enhance the replication of a different serotype during a subsequent infection. ADE can lead to more severe forms of dengue, such as dengue hemorrhagic fever or dengue shock syndrome [7,8,9]. Vaccine developers need to address this issue by ensuring that their vaccine candidates induce a strong neutralizing immune response to all serotypes without eliciting ADE. This requires careful selection and design of vaccine antigens to ensure they are effective against all serotypes and rigorous testing in preclinical and clinical studies. Furthermore, due to the hyperendemicity of dengue, vaccine developers also need to consider the potential for viral evolution and the emergence of new serotypes or variants [9]. Monitoring and surveillance efforts play a crucial role in understanding the epidemiology of circulating dengue serotypes and informing vaccine development strategies. Ongoing research and development efforts aim to overcome these challenges and improve prevention and control measures for this widespread and debilitating disease.

### 3.4. The Pathophysiology of Dengue Infection

Dengue infection comes along with various clinical manifestations ranging from mild, neglectable fever to severe physiological conditions. In the case of primary infection, only a mild level of disease develops, which is called DF [21]. It is characterized by mild headache, myalgia, fever, joint pain, abdominal pain, body pain, and retroorbital pain, along with immune dysregulation in the form of thrombocytopenia, lymphadenopathy, and leukopenia [6,21]. In the case of DHF, a high-grade hemostasis malfunction occurs. Moreover, severe vascular leakage often results in DSS, which is more harmful to patients. In DSS, patients undergo hypovolemic shock with reduced peripheral perfusion, leading to tissue injury, and, in more severe cases, it may result in multiorgan failure [6,8].

### 3.5. DENV-Host Immune Interactions (Innate and Adaptive Responses)

DENV regulates the host-cellular mechanism for manufacturing its further progeny. However, this cellular takeover is coupled with various challenges in the form of immune defense responses to viral progeny during various steps of the life cycle: viral entry, replication, and release [1]. DENV, however, actively escapes immune surveillance and often targets intracellular antiviral signals. Innate immunity induction begins with pattern recognition receptor (PRR) binding to pathogen-associated molecular patterns (PAMPs). In the first-line response of innate immunity, interferons (IFNs) and interleukins are produced from the signals of infected host cells, such as dendritic cells (DC). Similarly, natural killer (NK) cells also activate IFNs to quickly clear viral loads from the host body.

Toll-like receptors (TLRs) provide nonspecific innate immunity but play a vital role in detecting foreign DENV sensors that collaborate with the reactions of various immune cells [1,13,22]. Toll-like receptors (TLRs) are a family of pattern recognition receptors present in various immune cells, including dendritic cells, macrophages, and natural killer cells. These receptors play a crucial role in innate immunity by recognizing and responding to pathogen-associated molecular patterns (PAMPs) present on the surface of invading microorganisms [1]. In the case of dengue virus infection, several TLRs have been implicated in the innate immune response against the virus. TLR3, TLR7, and TLR8 are known to be involved in the recognition of viral RNA, which is a key component of the dengue virus [13]. These TLRs are specifically localized in endosomes, where they can interact with viral RNA. TLR3 recognizes double-stranded RNA, a common byproduct of viral replication. When TLR3 binds to viral RNA, it triggers downstream signaling pathways that result in the production of pro-inflammatory cytokines and type I interferons, which are essential for antiviral defense. Studies have shown that TLR3 activation can induce an antiviral state, limiting dengue virus replication in infected cells [13,22].

TLR7 and TLR8 are capable of recognizing single-stranded RNA, which is another form of viral RNA produced during the dengue virus infection. Activation of TLR7 and TLR8 also leads to the generation of pro-inflammatory cytokines and type I interferons, enhancing the antiviral immune response against the dengue virus. Furthermore, TLRs can collaborate with other innate immune receptors to mount a robust immune response to the dengue virus [22]. For instance, TLR3 can synergize with TLR4 and TLR7 to amplify the production of antiviral cytokines, strengthening the immune defense against dengue infection. In summary, Toll-like receptors such as TLR3, TLR7, and TLR8 play significant roles in the innate immune response against dengue virus infection. Their activation triggers downstream signaling pathways that result in the production of antiviral cytokines and interferons, limiting viral replication and facilitating the elimination of infected cells [1,13,22]. Understanding the role of TLRs in innate immunity against dengue may provide opportunities for the development of novel therapeutic approaches to this viral infection.

Several cellular signaling networks are activated upon receiving signals from released molecules. These pathways include activation of NF-κB, IK kinase-1 (IKK1)-associated phosphorylation of IRF3, myeloid differentiation primary response protein 88 (MyD88) and MAPK, activation of IFN-α/β, interferon-stimulating genes (ISGs). These molecules together lead to wide-scale stimulation and release of IFNs and cytokines of various types in the cellular atmosphere [22,23,24]. Other cellular entities, such as cytoplasmic helicases (retinoic acid-inducible gene I (RIG-I) and melanoma differentiation-associated gene 5 (MDA5), also play an important role in innate response generation against DENV. IFN-α/β causes inhibition of DENV infection by activation of the JAK-STAT signaling network, which plays a major role in the inhibition of viral infection [22,23,24]. Adaptor molecules such as TYK2, JAK1, STAT1, STAT2, STAT3, and STAT5 all play important roles in the activation of further networks such as the mitogen-activated protein kinase p38 cascade and the phosphatidylinositol-3-kinase cascade. These signaling networks then undergo the production of numerous antiviral proteins, proinflammatory cytokines, leukocytes, chemokines, and other antiviral entities inside the host cell [1,2,13,22,23].

### 3.6. DENV Strategy to Evade Innate Immune Responses

DENV displays several methods through which it interferes and averts host antiviral responses. It causes an extensive rearrangement of cellular membranes, along with modifications to host metabolism for progeny production. It inserts itself into cellular vesicles that remain unnoticed by host cell lysosomes and averts ER stress-induced responses [25]. DENV primarily uses the process of autophagy to replicate its genome. Moreover, it causes inhibitory responses toward multiple signaling cascades, IFN-linked networks, and RNAi pathways. DENV-specific proteins, such as nonstructural protein 4B (NS4B), NS2B-NS3 protease, and subgenomic flavivirus RNA (sfRNA), are currently under study to explain their critical role in hampering host antiviral responses in different mechanisms [20,23,25].

Moreover, DENV often mimics the cellular mRNAs of host cells and thus evades innate immune signals in the form of signaling cascades and IFN induction. Viral NS2A, NS4A, and NS4B also play an effective role in averting the JAK/STAT network and transcriptional activation of various host defense genes [24,26]. Instead of innate immune system responses toward viral loads, DENV causes direct ubiquitination and proteasome-mediated degradation of important cellular entities to avert innate immune responses in host cells. These averted responses further cause deviations in the adaptive immune response and overall disease outcomes [24,26].

### 3.7. Adaptive Immune Response to DENV

After unlabeled and ineffective innate immune responses, adaptive immunity sets up a line of defense in which both cellular and humoral responses are generated within 6–7 days after the first viral infection. Antibodies are released after the detection of DENV antigens on the surface of antigen-presenting cells (APCs), such as CD4+ T lymphocytes [20]. These antibodies are specific for the DENV envelope protein E and PrM glycoproteins on its surface. Studies have elaborated that nonstructural and structural proteins are differently recognized by CD8+ and CD4+ T cells, respectively [20,23,25]. This step causes the activation of adaptive immunity in the form of effector and helper T cells, which further undergo Th1- and Th2-mediated humoral immune responses in virus-infected cells. Th1 cells specifically induce inflammatory responses and tissue injury through IL-2, IFN-γ, and TNF-β. Th2 causes T-cell activation and proliferation through the secretion of IL-4,-IL-5, -IL-6,-IL-10,-IL-13, and other interleukins [27,28]. B-cells specifically regulate antibody production against viral proteins such as the NS1 protein. These antibodies cause the lysing of DENV-infected cells with the help of the complement system of adaptive immunity. Multiple other cellular interactions involving DENV NS1 protein, macrophages, PBMCs, TLR4, platelets, apoptotic molecules, NF-κB, and other signaling interactions mediate adaptive responses toward DENV [7,26].

It is important to understand the unbalanced production of cytokines, chemokines, and interleukins, which majorly contribute to the pathogenesis and clearing of DHF in an infected individual [1]. Finally, the viral epitopes interact with memory T cells, which undergo pro-inflammatory cytokine production and leakage through the vascular endothelium. The released material contains elevated levels of cytokines, chemokines, interleukins, and soluble CD4 and CD8 cells, which promote DHF and DSS in host bodies [23].

### 3.8. Treatment and Management of Dengue

**Figure 1:**
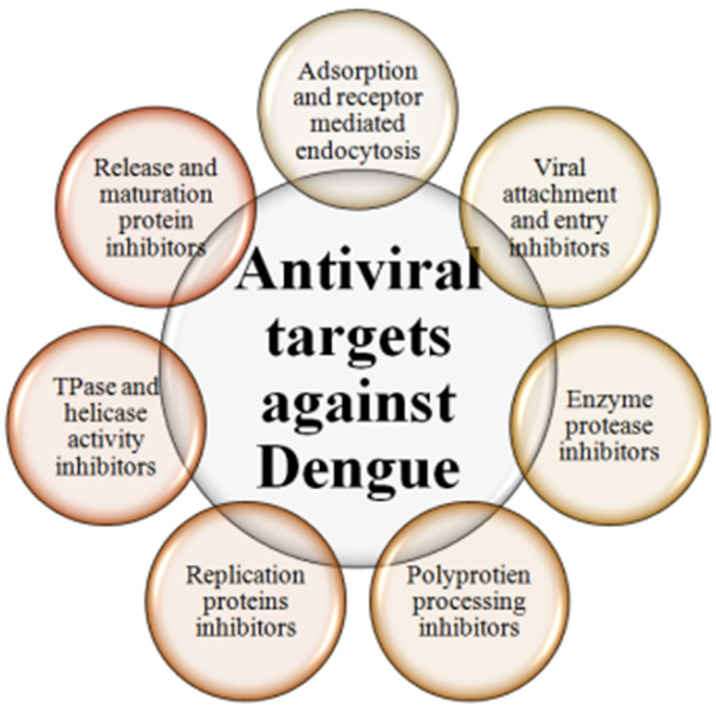
Antiviral and therapeutic targets Against DENV.

To date, no specific antiviral drugs have been approved for dengue virus treatment. However, several therapeutic options have been proposed, and some are being used to reduce infection outcomes. Several sulfated polysaccharides and antipyretics are in use against dengue because of their observed and approved antiviral properties [13,23]. Some specific polysaccharide compounds driven from seaweeds, including Curdlan, Caulerpa cupressoides, carrageenan G3d, and the dL-galactan hybrid C2S-3, have shown antiviral properties against all infectious serotypes of DENV [29]. They work by inhibiting host-viral interaction at receptor sites. Similarly, vaccine candidate ribavirin has been tested in combinatorial drug protocols and has been found to reduce DENV activity in host cells. Ribavirin is a guanosine (ribonucleic) analog used to stop viral RNA synthesis and viral mRNA capping [29,30]. Thus, ribavirin acts as a nucleoside analog and is used as a prodrug, which, when metabolized, resembles purine RNA nucleotides. In this form, it interferes with the RNA metabolism required for viral replication. Different mechanisms have been tested with ribavirin to check its efficacy as a prodrug against dengue. Derivatives of glycyrrhizin, nucleoside adenosine, NITD008, and uridine analog 6-aziridine-based experiments exhibited anti-DENV properties as they work by modifying and inhibiting DNA and respective protein synthesis [31,32]. Moreover, some recent studies have also indicated treatment options based on CP26, CDDO-me, UV-4B, ivermectin, and ketotifen for controlling dengue outbreaks in the future [32,33]. Some basic therapeutic target approaches against dengue have been compiled in Figure 1 to show the major mechanisms of therapeutic development and targets against dengue.

### 3.9. The Development of Vaccines against Dengue

The sustained nature of DENV and its associated diseases, which have shown a healthcare burden over the years, implicates the production of antidengue vaccines against them. The control of the vector population and preventive public measures are also important in this regard. Genomic mutations in the viral genome and drug resistance are the major obstacles in the process of vaccine development. The mutational genomic character can be easily observed in the DENV outbreaks of 2006 and 2011 [23,25]. The high mutation rates aiding in drug resistance development in DENV present a greater threat to the healthcare system. Therefore, the vaccination and drug development processes should be managed at the same pace. Mice and nonhuman primates are being used as animal models for checking anti-DENV vaccine candidates. Different vaccine candidates, such as tetravalent vaccine formulations, whole-virion vaccines, synthetic peptides, subunit vaccines, recombinant live vector vaccines, infectious cDNA clone-derived vaccines, and naked DNA-attenuated viral vaccines, are being proposed for treating DENV infection. In the following section, we will discuss specific vaccination protocols and respective drug trials against DENV that have been in practice and are under further investigation by different laboratories [9,15,34,35,36].

### 3.10. Targets and Strategies for Vaccine Development against Dengue Viruses

**Figure 2:**
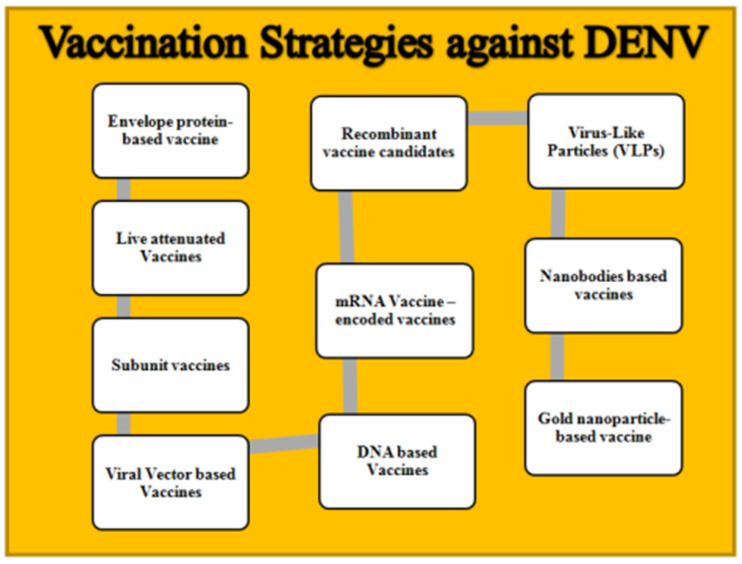
Vaccination strategies against DENV.

A brief overview of different vaccination strategies that are in various trial phases against DENV has been included in Table 1, with a figurative representation in Figure 2. Different therapeutics options for dengue virus are shown in Table 2.

### 3.11. Vaccine Candidates in Advanced Clinical Trials

Recently, live-attenuated tetravalent DENV vaccine candidates, namely Sanofi Pasteur CYD-TDV (Dengvaxia/ChimeriVax candidate), DENVax, and TV005, are undergoing phase II clinical trials and are expected to be processed for phase IV trials soon [7,30,49] The WHO, and FDA have approved Dengvaxia to be implemented in US territories against all serotypes of the dengue virus. Another promising vaccine candidate is DENVax (live-attenuated dengue vaccine), which originated from the Centers for Disease Control and Prevention of the USA (CDC, USA). It was renamed TAK-003 after getting licensure at Takeda (Japan) [7,9,30]. TAK-003 actively works against four serotypes of DENV and maintains immunity for up to 48 months without causing disease severity. Overall, TAK-003 shows ~80% efficacy against DENV infection [35]. A tetravalent vaccine candidate (TV005) is another interesting development in DENV vaccine development. It is based on a modified strain of the DENV genome that caused immunogenicity in the tested subject. It has shown effective antibody-mediated immune responses in 90% of checked subjects [31]. Other vaccine formulations against DENV include a live attenuated tetravalent vaccine for dengue (LATV), recombinant DNA vaccine, tetravalent dengue (TDEN), dengue purified inactivated vaccine (DPIV), tetravalent DNA vaccine against dengue (TVDV), tetravalent vaccine formulation (V180) with enveloped glycoproteins, and D1ME100 DNA vaccine, all of which are in experimental phases [6,31,51].

### 3.12. Antibody-Dependent Enhancement (ADE)—The Main Obstacle for Vaccine Development against Dengue Virus

Antibody-dependent enhancement (ADE) is a phenomenon in which certain antibodies, instead of neutralizing them, actually enhance the entry of a virus into target cells and facilitate its replication [15,52]. This can occur when non- or subneutralizing levels of antibodies are present during a subsequent infection with a related but different strain of the same virus. In the case of dengue virus, there are four different serotypes (DEN-1, DEN-2, DEN-3, and DEN-4) [31,36]. A primary infection with one of these serotypes usually leads to long-lasting protective immunity against that particular serotype. However, subsequent infections with a different serotype can lead to more severe disease due to ADE.

The molecular mechanisms behind ADE involve the interaction of antibodies with specific cell receptors called Fc receptors. During a secondary infection, antibodies produced in response to the primary infection bind to the new infecting serotype but fail to neutralize it completely [15,52]. Instead, these antibodies facilitate viral entry into immune cells, such as monocytes or macrophages, through their interaction with Fc receptors on these cells. This enhances viral replication and can lead to a more severe and potentially life-threatening disease. ADE can occur due to several factors, including the presence of non-neutralizing antibodies that bind but cannot fully neutralize the virus, the presence of cross-reactive antibodies that are more effective in binding to the new serotype, or the presence of higher levels of antibodies that can overwhelm the neutralizing capacity [15,34,52].

ADE poses a major obstacle to the development of a dengue vaccine, as it is important to induce a protective immune response without eliciting ADE. Vaccine candidates must ensure that the induced antibodies are highly neutralizing and do not enhance viral entry [9,31,34]. Additionally, vaccines should elicit a balanced immune response to multiple dengue serotypes to reduce the risk of ADE during subsequent infections. Researchers and vaccine developers are working on strategies to overcome ADE in dengue vaccine development. This includes the use of carefully designed vaccine antigens that elicit a strong neutralizing antibody response and minimize the generation of cross-reactive antibodies. Alternative vaccine platforms and adjuvants are also being explored to enhance the production of neutralizing antibodies while minimizing ADE. In summary, ADE-based molecular mechanisms pose a significant challenge in dengue vaccine development [7,9,31,35]. Overcoming ADE requires the induction of highly neutralizing antibodies that can effectively prevent viral entry without facilitating it. Extensive research and development efforts are being undertaken to tackle this obstacle and create safe and effective dengue vaccines.

## 4. Future Directions

Over the past three years, the scientific community has become increasingly concerned about infectious diseases due to the pandemic era. This is the very reason that so much research is being conducted on viral diseases, such as dengue, Ebola, COVID, HBV, HPV, HIV, and Zika [46,53]. There is a need to look into modern therapeutic approaches, such as those based on nanotechnology, to bring out-of-the-box solutions for vaccine and drug development. In developing nations, special focus should be kept on adaptive strategies to cope with the dengue virus [46,53,54,55]. The available and already established vaccination protocols should be checked for further confirmation. Moreover, some of the latest technologies, such as plant-based organic compounds and nanomedical applications, should be incorporated within the available vaccination and therapeutics development drives to create improved vaccination and medication protocols [12,55,56,57,58]. In the future, standard methods of vaccination, such as live attenuated viruses and DNA viruses, will be advanced in terms of manufacturing techniques, such as a dual application of genetic engineering and nanobiotechnology, which may be useful to improve therapeutic manufacturing, dosages, outcomes, and clinical experimentation compared to previous drug protocols [5,59]. 

## 5. Conclusions

A comprehensive overview has been presented in this review article for the readers to understand the gaps remaining in the medication and vaccination journey against dengue infection. No approved antiviral drug is available for the treatment of dengue infection. There are many vaccine candidates for the dengue virus at different stages of development, but only two vaccines have been approved for public use. These vaccines are effective but have limitations. There is a need to increase efforts to develop new vaccines for dengue. 

## Figures and Tables

**Table 1:** Different vaccination strategies against DENV.

Sr. no.	Vaccine Strategy	The Mechanism of Action and Vaccine Development	Vaccine Candidates under Trials	References
1	Envelope protein-based vaccine	Structural viral antigens directed at envelope proteins (E and M of DENV) avert the viral-host interaction and their underlying activation responses that are essential for viral genomic regulation. The E protein is the structural regulator of DENV and plays a vital role in DENV-associated pathogenicity and vaccine development. However, the associated nonspecific immunoglobulin production is a major challenge to overcome for vaccine designing.	E85-VRP and prM-E-VRP Modified vaccinia ankara (MVA) virus—E proteins Venezuelan equine encephalitis virus replica particles (VRP) infused M and E proteins.	[2,7,13,23,27,28]
2.	Live attenuated vaccine candidates	Nonstructural proteins of DENV with important roles in the replication cycle are used to design attenuated vaccination protocols with controlled virulence and enhanced immunogenicity. More specifically, the envelope E and NS1 proteins mediate complement-fixing (CF)-induced antibody responses in the host immune system. Similarly, recombinant DENVs are modified either by deletion or antigenic chimerization to generate attenuated vaccines against all four serotypes of DENV through reverse genetics.	DENV2 NS1. DENV2-E and NS1 fused to a staphylococcal A protein. Tetra-live attenuated virus (TLAV). DENV-2 NS1 recombinant proteins – + adjuvant (alum) and Freund’s adjuvant (FA). Tetra DIIIC. DENV2 16681 mutant strains. DENVax-based vaccines. tetravalent dengue vaccine, CYD-TDV (Dengvaxia®) Takeda’s tetravalent TAK-003 (DENVax) TV003/TV005 (NIH)	[1,3,4,6,8,13,17,27,37,38,39]
3.	DNA vaccine candidates	The use of plasmid DNA for encoding different viral proteins, such as the DENV-NS1 protein, promulgates an immunogenic response. Moreover, the plasmids can be loaded with immune system cells such as interleukin-12 that help further elicit a stronger immune response. Various adjuvants are being tested with DNA vaccines to increase the immunogenicity of future vaccine candidates.	DENV-2 plasmid, pEII*EIII/NS1 DENV2 NS1 fused to a t-PA signal (NS1-tPA). DENV1 E80 D1ME100 DENV2 prM/E - CpG motif DENV2 prM/E DNA Tetravalent DNA Vaccine (TVDV) (Vaxfectin®)	[3,4,8,13,17,27]
4.	Vector vaccine candidates	Virus vector candidates are used for expressing desired genes such as NS1, NS2A, and protein derivatives for all four serotypes of DENV. These vectors undergo similar viral responses in a host cell, such as glycosylation, and sedimentation. The viral species with low pathogenicity and good deliverability, such as vaccinia virus, adenovirus, and alphavirus, are mostly employed.	DENV4 NS1 and NS2A recombinant vaccinia virus. Baculovirus-expressed NS1 candidate. Baculovirus - DENV-4 NS1- (C-M-E-NS1-NS2a). Modified vaccinia Ankara (MVA) virus - E proteins EDIII domains Adenovirus - based recombinant dengue virus − 1 and − 2 (CAdVax-Den1–2) Dengue virus − 3 and − 4 (CAdVax-Den3–4) Venezuelan equine encephalitis virus replicon particles (VRP)- infused M and E proteins E85-VRP and prM-E-VRP	[4,6,8,13,14,29,35,38]
5	Subunit vaccine candidates	Ease and cost-effectiveness. The respective studies show short- to long- term immunity induction through high titers of neutralizing antibodies against different DENV stereotypes.	DENV2 rEDIII.	[8,27]
6.	Recombinant vaccine candidates	Includes the fusion of E proteins with lipoproteins, which undergo antigenic representation and respective immune responses in the host.	Tetravalent lipidated rEDIII formulation. DENV1-rEDIII linked with DENV2-rEDIII DENV3-rEDIII linking with DENV4-rEDIII Tetravalent subunit vaccine V180	[6,8,9,13,16,27]
7.	Inactivated vaccine candidates	Several protein entities, such as C, M, E, and NS1, are used for designing antigenic components in inactivated vaccine candidates. Pathogens and antigenic substances are treated with several chemicals and radiations to reduce their infectivity in subjects. These vaccine candidates possess a lower risk of infection activation.	DENV2 vaccine (S16803) S16803 vaccine-associated drug adjuvants (including alum, AS04, AS05, and AS08) Recombinant vaccine S16803- (R80E) and LAV (DENV2 PDK-50) Tetravalent purified formalin-inactivated virus (TPIV) or tetravalent DNA vaccine (TDNA) – coupled with tetravalent live attenuated vaccines (TLAV) Dengue purified inactivated vaccine (DPIV)- AS03_B_ adjuvant Tetravalent purified formalin-inactivated virus (TPIV).	[16,31,40,41]
8.	mRNA vaccine encoded vaccines	Some vaccines are designed on the basis of lipid nanoparticles (LNPs) encapsulated with modified mRNA and DENV1-NS. The serotype-specific immune responses could be generated by properly designing mRNA-LNP vaccine candidates.	DENVI-NS vaccine prM/E mRNA-LNP vaccine	[42,43]

**Table 2:** Some novel therapeutic approaches against DENV.

Sr. no.	Therapeutic Strategy	Explanation	Candidates under Trials	References
1.	Designing of nanobodies against dengue virus proteins	Some available nanobodies are being restructured and repurposed for the treatment of DF. The nanostructures are being conjugated at antibody regions to deliver drug adjuvants against infectious viral agents. Moreover, nanoassemblies are also being progressively employed for diagnostic purposes for viral detection in patients.		[42,43,44,45,46]
2.	Gold nanoparticle-based subunit vaccine	Gold nanoparticles combine different structural components of DENV, such as enveloped glycoproteins, through hybrid technology. The results generated have shown that AuNP-E induced a high level of antibody-mediated neutralization against DENV. Scientists believe that by controlling the size and dosages of AuNPS, more stimulatory responses could be produced.	AuNP-E subunit vaccines. Tetravalent AuNP-based subunit dengue vaccine.	[43,47,48]
3.	Polymerase inhibitor—based drugs	Selectively inhibits iral RNA-dependent RNA polymerase without creating cytotoxicity in mammalian cells.	T-705	[30,49]
4.	Promising therapeutic candidates derived from natural products	Some new studies are being conducted to extract natural resources for dengue treatment. However, there is a need to further work on these pharmaceuticals to establish their anti-DENV drug properties.	1,25 dihydroxy vitamin D_3_ (1,25(OH)_2_D_3_), the active form of vitamin D Curcumin (termaric) Betulinic acid	[33,44,50]

## Data Availability

Not applicable.
